# Trauma exposure and PTSD prevalence among Yazidi, Christian and Muslim asylum seekers and refugees displaced to Iraqi Kurdistan

**DOI:** 10.1371/journal.pone.0233681

**Published:** 2020-06-24

**Authors:** Sami Richa, Marie Herdane, Azzam Dwaf, Rami Bou Khalil, Fadi Haddad, Rhéa El Khoury, Myriam Zarzour, Anthony Kassab, Ramez Dagher, Alain Brunet, Wissam El-Hage

**Affiliations:** 1 Faculty of Medicine, Hôtel-Dieu de France, Saint Joseph University, Beirut, Lebanon; 2 McGill University/Douglas Mental Health University Institute, Montréal (Qc), Canada; 3 UMR 1253, iBrain, Université de Tours, Inserm, CHRU de Tours, Clinique Psychiatrique Universitaire, Tours, France; Uniformed Services University of the Health Sciences, UNITED STATES

## Abstract

**Background:**

There is unreliable, and negligible information on the mental health and trauma-exposure of asylum-seekers and displaced refugees in the Iraqi Kurdistan region.

**Objectives:**

To evaluate how responsible the ethno-religious origins are, for the prevalence of trauma exposure and post-traumatic stress disorder (PTSD) in displaced Iraqi asylum-seekers and refugees residing in the Iraqi Kurdistan region.

**Methods:**

Structured interviews with a cross-sectional sample of 150 individuals, comprised of three self-identified ethno-religious groups (50 participants in each): Christians, Muslims, and Yazidis.

**Results:**

100% prevalence of trauma exposure and 48.7% of current PTSD among refugees, 70% PTSD rate of Yazidi participants, which is significantly higher (*p* < 0.01) compared to 44% of Muslim participants and 32% of Christian participants. These findings were corroborated using the self-rated PTSD, DSM-5 Checklist, with more severe PTSD symptom scores (*p* < 0.001) obtained among Yazidis (43.1; *19*.*7*), compared to Muslims (31.3; *20*.*1*) and Christians (29.3; *17*.*8*). Self-rated depressive symptoms (Patient Health Questionnaire-9) were also higher (*p* < 0.007) among Yazidis (12.3; *8*.*2*) and Muslims (11.7; *5*.*9*), compared to Christians (8.1; *7*).

## I. Introduction

In 2016, the United Nations High Commissioner for Refugees disclosed the highest number of refugees since 1996, with the population of concern also including asylum seekers and internally displaced persons, standing at 65.6 million up from 46.3 million at the midyear of 2014 [1]. Middle-Eastern populations have been increasingly exposed to war, and victims of persecution and human rights violations in the midst of long-lasting political conflicts. Since 2003, these events have caused several waves of migration, particularly from Syria and Iraq [2,3]. Iraq has consistently scored 5 on the Political Terror Scale, making it one of the seven worst offenders of human security. According to the 2013 U.S State Department report, societal abuses and discrimination are based on religious affiliation, belief, or practice. In fact, ISIS rebels, motivated by a confluence of religious, political, and cultural factors, are threatening, oppressing and murdering those who do not conform to their religious ideals, including Yazidis, Christians, and Muslims who do not adhere to their ideology.

The Yazidi religion is considered a pre-Islamic sect that draws from Christianity, Judaism and the ancient monotheistic religion of Zoroastrianism. At the core of the Yazidis’ marginalization is their worship of a fallen angel, who is often misunderstood to be Satan by other religious groups. They have consequently faced multiple genocides in their history, during Saddam Hussein's regime. In August of 2014, the terrorist organization ISIS attacked the largest Yazidi community, of approximately 400,000 people, who were living in the Mount Sinjar area, forcing them out of their homeland and fleeing to safer places.

In the last five years (since 2014), the Christian population in Iraq has declined by nearly 300,000 [4]. Indeed, after the fall of Mosul, more than 100,000 Christians fled Nineveh and streamed into the relatively safe Kurdish region. They found refuge in Irbil and Dohuk, in camps managed by humanitarian organizations and churches. The Nineveh Reconstruction Committee, an ecumenical partnership between the Chaldean Catholic Church, Syriac Catholic Church and Syriac Orthodox Church, restored thousands of houses in two towns, allowing more than 11,000 displaced Christians to return home.

Despite the risk ISIS poses to Yazidis, Christians and other minorities in the country, the risk to Iraq's majority Muslims is far more widespread; most of the 4.2 million displaced Iraqis are Sunnis, adherents to the branch of Islam that ISIS claims to represent. In fact, as the battles intensified to capture Mosul, Iraq’s biggest Sunni city, and Raqqa, the group’s self-proclaimed capital in Syria, many Sunni towns were being destroyed and depopulated; Sunnis were left with no possessions and profoundly marked by ISIS militant’s atrocities. They chose to seek shelter in Kurdish-controlled provinces, thus overcoming cultural and linguistic differences as well as political conflicts between the Iraqi and the Kurdish governments. Mainly Muslims were made responsible for the ethnic imbalance in those provinces, making it harder for their socio-cultural integration.

Asylum seekers and refugees are often exposed to traumatic events and psychosocial stressors (e.g., extreme violence, separation from family or detention in a concentration camp), not only before and during displacement, but during the post-displacement period as well, after they are resettled in suboptimal conditions in the host country [5–8]. Prevalence of Post-traumatic stress disorder (PTSD) diagnostic in refugees varies considerably, due to life circumstances of the sampled individuals, as well as the instruments and methods used [9,10]. A positive association is frequently found between pre-migration/migration potentially traumatic events and PTSD or depression [11–16]. Given their exposure to torture, trauma and mass displacement, Iraqi refugees living in Turkey report a high prevalence of PTSD of 55% [17,18]. During the last 5 years, a considerable amount of changes has occurred in the Iraqi population [4,19–22]. This data does not however take into consideration the difference in symptomatology between the different religious groups, and more data is required regarding the impact many factors have on the mental health of refugees and more precisely, the cultural, religious and overall background of the refugees, in order to better asses any potential role these factor will have on the psychiatric symptoms. The multicultural and religiously complex aspect of the refugee crisis In Iraq makes it possible to take into account all of those factors.

The objective of our study was thus to evaluate the levels of PTSD and depressive symptoms in the Iraqi refugee population by assessing and comparing the prevalence of symptoms among different confessional groups of refugees, residing in Iraqi Kurdistan.

## II. Material and methods

### II.1 Ethical considerations

The study’s protocol, has been approved by the ethics committee of Hôtel-Dieu de France university hospital in Beirut (reference number: 882).

### II.2 Participants

This study was conducted starting 11/2016 and ending 01/2017 at the Erbil refugee camps, that accommodate Iraqi refugees from different religious beliefs and socioeconomic characteristics, although most of the refugees who seek help in the camps belong to a low socio-demographic status. All specific groups are welcomed within the camps and are subject to equal rights and obligations irrespectively of their confession and cultural background. Sample groups visited by a team of social workers were consecutively selected. All Iraqi refugees in the camp are systematically and frequently visited by social workers regardless of their distress level or any need. As a matter of fact, recruitment was made by a group of social workers that agreed to participate as investigators in this study. In their routine assessment and for three months, they selected all eligible Iraqi refugees that could participate in the study. Inclusion and exclusion criteria were, refugees between the ages of 18 and 65, adherents to Christian, Muslim and Yazidi communities, fluent in Arabic language, with no psychiatric disorders (including PTSD), currently not being treated for any psychiatric disorder, and participating in no other studies. A total of 360 refugee were approached in this period of time, of which 180 were considered being eligible to be included in the protocol. The sample was approximately homogenous in terms of confession. Then, this sample was divided in three groups, according to the three different confessions (Christianity, Yazidi, and Islam). The first 50 participants of the same confession having provided written, fully anonymous informed consent were included in the same group. Thus, a total of 150 participant was selected, divided in three groups of 50 according to the three different confessions. To be noted that the number of each religious group was representative of the overall makeup of the camp.

### II.3 Assessment instruments

With the cooperation and permission of the Erbil Jesuit Refugee Service (JRS) head, a duo of a psychotherapist and a specifically trained general practitioner, visited the camps on a regular basis, conducting interviews under appropriate mental examination conditions with the 3 selected groups of participants as described earlier. A psychiatrist confirmed the cases that were screened via the questionnaires.

Data was collected using a socio-demographic history form in Arabic language (used in the camps), including age, sex, education level, marital status, number of children, religion, occupation, monthly income, household crowding, duration of displacement, as well as psychiatric and other chronic organic diseases.

The Life Events Checklist (LEC) is a 17-question self-report measure, that captures trauma types as compared to the frequency of traumas. It was used to assess potentially traumatic events exposure, on a 5-point nominal scale and administrated alongside with the PTSD Checklist for DSM-5 (PCL-5) [19].

PCL-5 is a 20-item self-assessment questionnaire, that measures PTSD symptoms, (during the past 30 days) as listed in the DSM-5 according to a 0 to 4 rating scale; for each item, a score of 2 or above is regarded as clinically relevant. A PCL-5 cut-point of 33 is recommended to consider PTSD. The 20-item self-rated PCL-5 was used to assess PTSD symptom severity in the last month. We used the interviewer-rated MINI (Mini International Neuropsychiatric Interview) to confirm the PTSD diagnosis [23]. Depression’s high prevalence in this clinical setting and its high comorbidity with PTSD, we screened for it, by using the 9-item self-report Patient Health Questionnaire (PHQ-9) depression subscale. With 9 items, this instrument is half the length of many other depression scales, with a comparable sensitivity and specificity making it a reliable measure for depression severity. It scores each of the 9 DSM-5 criteria as 0 (not at all) to 3 (nearly every day) [24]. All questionnaires that were used were validated in Arabic.

Crowding index is an established and defined tool used for the evaluation of socioeconomic status in a study population, with a negative association with socioeconomic characteristics. It is defined as the total number of co-residents per household, with the exclusion of the newborn infant, divided by the total number of rooms, excluding the kitchen and bathrooms [25]. All instruments were culturally and linguistically adapted to suit the populations of interest. While screening instruments were used for this study, it is important to point that no PTSD diagnoses were made without a psychiatrist’s clinical assessment.

### II.4 Statistical analysis

Chi-square tests were performed to compare rates among the study groups, with the Fisher’s exact test being employed when one or more of the cells had an expected frequency < 5. Independent samples *t*-test were performed when comparing two group means of a normally distributed, homoscedastic interval dependent variable. Statistical significance was set at *p* < 0.05 in a two-sided test, for all tests. No measure was taken to control for alpha inflation due to multiple testing in this exploratory study. The statistical analysis was performed using SPSS (IBM Corp, New York) version 20.0.

## III. Results

### III.1 Characteristics of participants

Fifty participants were recruited from each of the three religious groups (Yazidis, Muslims and Christians). The socio-demographic data for each group is presented in Table 1. It can also be assumed that the refugees are from a low socio-economic status due to their presence in camps. No between-group differences were found in terms of mean age, gender or the presence of organic or severe psychiatric illness. A significant difference was observed between the Yazidis and the Christians, with the latter having more single participants than the former (*p =* 0.001). Moreover, a higher educational level was reported among Christians and Muslims in comparison with Yazidis (*p* < 0.001). Similarly, Yazidis had a lower monthly income (< 400$ vs. > 400$) compared to Muslims and Christians (*p* = 0.002 and 0.009, respectively). The Yazidi population obtained a significantly higher crowding index than the other two populations (*p* = 0.02 and < 0.001, respectively).

**Table 1:** Sociodemographic characteristics of participants by religious affiliation.

Variable		Group		
		Yazidis (*n* = 50)	Muslims (*n* = 50)	Christians (*n* = 50)
Mean age [range] in years		36.5 [20–65]	35.5 [19–59]	36.1 [16–60]
% (*n*)		% (*n*)	% (*n*)	% (*n*)	
Sex	Men	50 (25)	48 (24)	50 (25)
	Women	50 (25)	52 (26)	50 (25)
Civil status ^Δ Δ^	Single	10 (5)	22 (11)	38 (19)
	Married	90 (45)	74 (37)	62 (31)
	Divorced	0	4 (2)	0
Educational level ^Δ Δ^	Uneducated	54 (27)	10 (5)	4 (2)
	Primary	26 (13)	10 (5)	14 (7)
	Middle school	4 (2)	20 (10)	14 (7)
	Secondary	8 (4)	16 (8)	6 (3)
	University	8 (4)	44 (22)	62 (31)
Monthly income (US$) **	0–200	20 (10)	2 (1)	6 (3)
	200–400	22 (11)	10 (5)	12 (6)
	400–600	28 (14)	28 (14)	20 (10)
	> 600	30 (15)	60 (30)	62 (31)
Chronic organic disease	Present	8 (4)	10 (5)	8 (4)
	Absent	92 (46)	90 (45)	92 (46)
Severe psychiatric illness	Present	0	0	0
	Absent	100 (50)	100 (50)	100 (50)
Mean crowding index [*range*; *SD*] ^Δ^		3.1 [0.2–8; 2.3]	1.9 [0.3–5; 1.1]	1.5 [0.3–6; 1.4]

Statistical significance threshold

**p < 0.01

^Δ^*p* < 0.005

^Δ Δ^*p* < 0.001

### III.2 PTSD analysis

**Fig 1:**
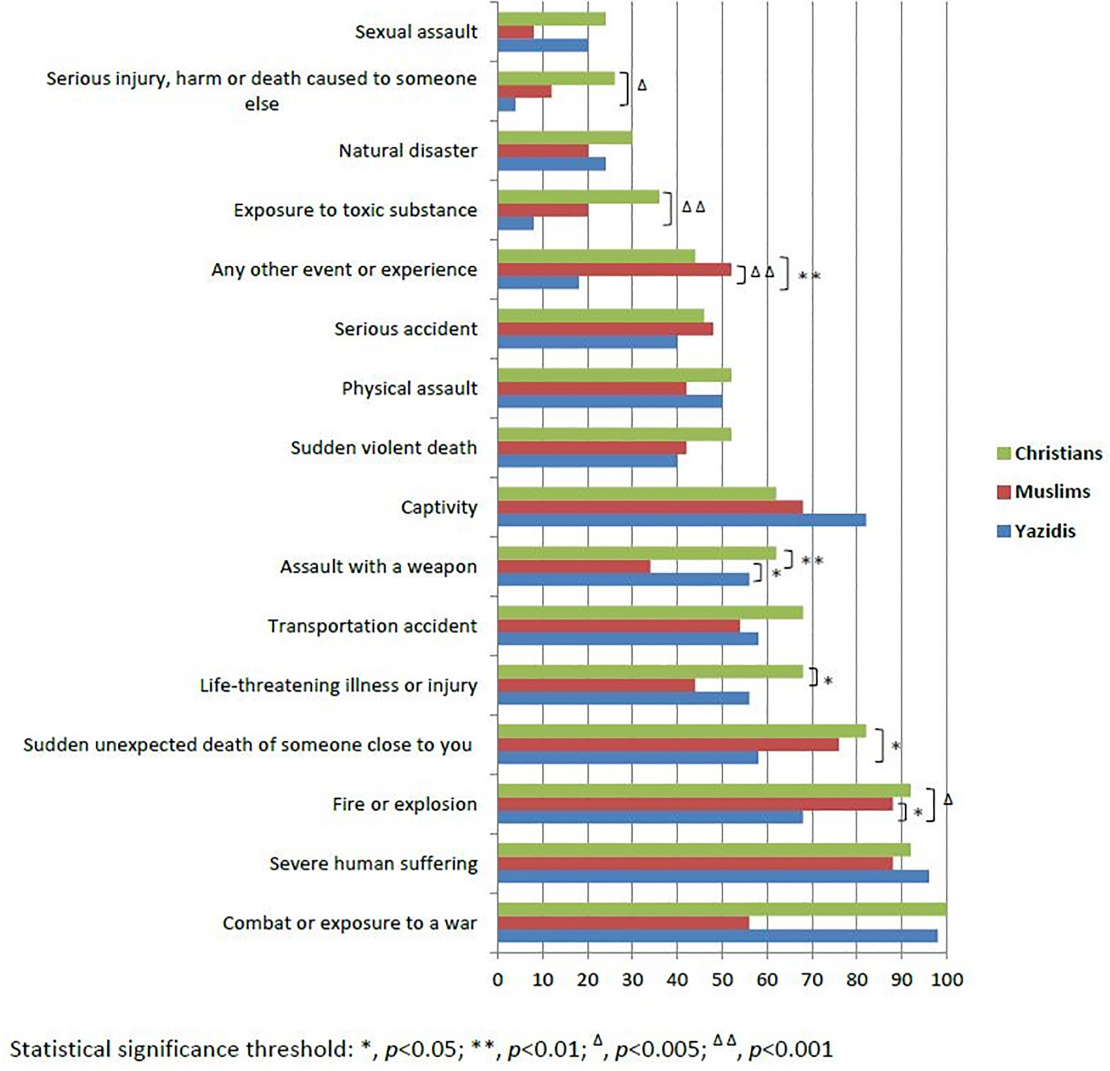
Lifetime trauma exposure according to religious affiliation.

Overall, based on the structured interviews, the prevalence of trauma exposure (defined as an exposure to at least one traumatic event, with or without a diagnosis of PTSD) among refugees, was 100%, and that of current PTSD was 48.7%. Fig 1 summarizes trauma exposure among the three religious’ groups. Christians were found to be the most exposed to trauma in general, as compared to Muslims and Yazidis.

**Fig 2:**
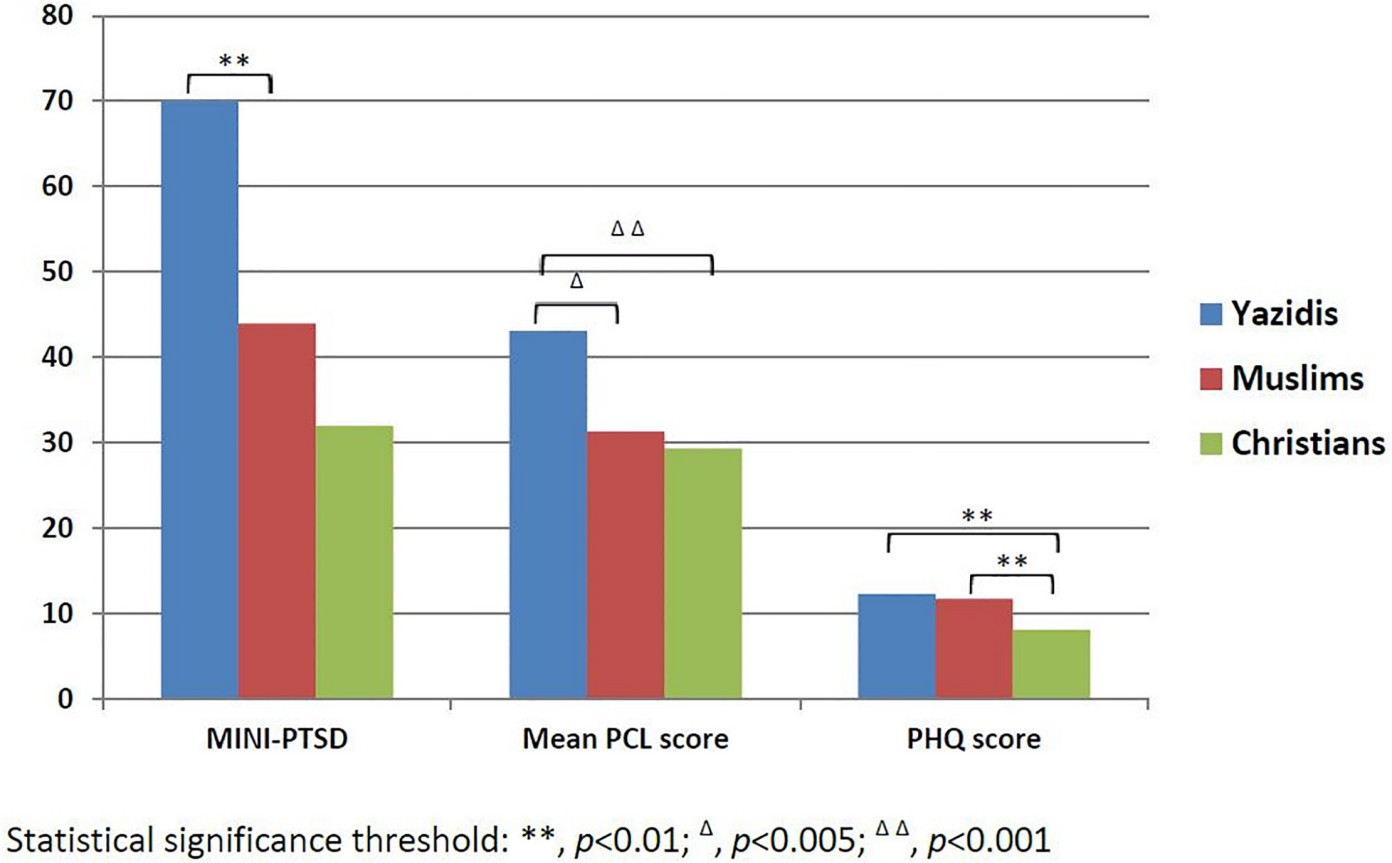
PTSD and depression scores according to religious affiliation.

As shown in Fig 2, the PTSD rate was found to be significantly higher in Yazidi participants, compared to Muslims (70% v/s 44%; 95% CI, 0.07 to 0.5; *p* = 0.008) and to Christians (70% v/s 32%; 95% CI, 0.2 to 0.6; *p*<0.001). Table 2 summarizes the difference in the PTSD symptom score and the depressive symptom score between the three populations. The mean PTSD symptom score (PCL-5) was higher among Yazidis (43.1; *19*.*7*), compared to Muslims (31.3; *20*.*1*) and Christians (29.3; *17*.*8*). The depressive symptom (PHQ-9) score was higher for Yazidis (12.3; *8*.*2*) and Muslims (11.7; *5*.*9*) compared to Christians (8.1; *7*), while no difference was noted between Yazidis and Muslims (*p* = 0.676). When analyzing the occurrence of PTSD in relation to age and gender, it was found that PTSD occurred more frequently in women compared to men with a ratio of 1.52:1 (*p* = 0.016). However, no correlation was found between age and PTSD. Furthermore, when assessing the difficulty of current problems, more Christians noted that their problems were “somewhat difficult” compared to Yazidis (*p* = 0.036); however, more Yazidis mentioned that their problems were “very difficult” compared to Christians (*p* = 0.039) and “extremely difficult” compared to Muslims (*p* = 0.036) and Christians (*p* = 0.036).

**Table 2:** Difference in PCL-5 and PHQ-9 scores between the three populations.

Religion	PCL-5				Religion	PHQ-9			
	Score	Mean difference	95% CI	p		Score	Mean difference	95% CI	p
Christian	29.3	13.9	6.4–21.3	<0.001	Yazidi	12.3	4.2	1.1–7.2	0.008
Yazidi	43.1				Christian	8.1			
		11.9	4–19.7	0.004			3.6	1–6.1	0.007
Muslim	31.3				Muslim	11.7			

## IV. Discussion

Within the internally displaced Iraqi population, all refugees reported a high rate of trauma exposure and of PTSD, before, during, and after displacement. Yazidis presented the highest rate of PTSD as compared to Christians and Muslims, regardless of trauma exposure. In addition, although Christians reported higher levels of exposure to traumatic war events, they were found to be suffering less from depressive symptoms than the Yazidis and Muslims.

PTSD prevalence rates in the setting of the internally and/or externally displaced Iraqi population fluctuate among studies. PTSD prevalence in Yazidi children and adolescents displaced to Turkey is 10% according to a retrospective review of detailed psychiatric interviews [12]. This prevalence was 42.9% according to structured clinical interviews conducted in a larger sample of Yazidi refugees from Turkey [3]. In the Iraqi displaced population resettled in western countries, the prevalence of PTSD ranges between 8–37% [9]. Accordingly, PTSD prevalence depends on several factors, such as the participants’ age, the level of exposure to war events, the resettlement situation, the screening tool used, etc. Due to the difficulties of randomization strategies, these factors are hard to control in studies including Iraqi refugees. However, with 70% of the Yazidi population scoring positively, it appeared to be the most vulnerable to PTSD, while having a mean PTSD symptom score of 43.1 on the PCL-5 [19].

In the current study, the LEC presented a broader spectrum of adverse events that are not specific to the war traumatic events, classically used by other studies on refugees. According to our study sample, Christians reported in their answers, having witnessed more “serious injury, harm or death caused to someone else”, more “exposure to toxic substances”, more “assaults with a weapon”, more “life threatening illness or injury”, more “sudden unexpected death of someone close” and more exposure to “fire or an explosion”. Although ISIS’s atrocities committed against the Yazidi community have been well documented and reported by the media, Christians still reported to have been exposed more frequently, to adverse life events, than the Yazidis. It can be speculated that several factors, such as education level and socio-economic status, related to the assessed populations, may have contributed to these findings.

Despite a lower exposure to traumatic experiences, Yazidis reported the highest PTSD risk rate, highly probable, due to post-resettlement circumstances and lower education level and socio-economic status. The Yazidis in our sample, had a lower monthly income and a higher crowding index, than those of Christians and Muslims. In addition, Yazidis mentioned that their problems were “very difficult” much more than those of Christians and “extremely difficult” more so than those of Muslims and Christians. The above sheds light on the importance of post-resettlement life circumstances, to be a significant predictor of PTSD development or its persistence. Post-migration stress, is an important factor in predicting the occurrence of mental disorders in refugees. Acculturation, which is the process of social, psychological, and cultural changes, stemming from the blending of cultures, appears to be one of the major contributing factors, to post-migration stress [26]. In the context of Yazidi refugees in Iraqi Kurdistan, acculturation can be explained due to the fact that most conservative Yazidis speak neither Arabic nor Kurdish, they do not mix with people of other religions, they can hardly find jobs, due to their low education level and the discrimination they might be subject to, in host communities.

Prevalence of depression in Iraqi refugees varies between 28.3 and 75% [9]. In our study, 56% of Yazidi refugees and 68% of Muslim refugees reported above the threshold of vulnerability to depression, as screened by the PHQ-9. Depression is often comorbid with PTSD in refugees. It is also related to known and significant pre-migration and post-migration stress. One of the most prevalent post-migration stressors in the resettled Muslim community in Iraqi Kurdistan, is related to the fact that Arab Muslim refugees have difficulty landing jobs, due to longstanding historical conflicts between Arabs and Kurds, and the discrimination it might generate. Another post-migration stressor, specific to the Muslim population that has fled the ISIS strikes, is related to the lack of enough psychosocial support, from international organizations, which were more focused on helping the supposedly more vulnerable religious minorities. As for the Yazidis, one of the major post-migration stressors that might be more specifically related to the development of depression, rather than any other mental disorder, are guilt feeling and shame, in more general terms, a “loss of pride” feeling [16]. In the Yazidi community, as in many other cultural and religious minorities throughout the world, a monumental collective identity component, is related to a strong attachment to the community’s geographical location and religious rituals. In Iraqi Kurdistan, the majority of displaced Yazidis, express the “loss of pride” feeling, because they have lost their religious identity, their historical roots, their cultural habits, through genocide and violence. In fact the genocide perpetrated by ISIS has resurrected in the Yazidis memories 74 of their ancestors’ massacres across 800 years. As a result, the “loss of pride” feeling, emanates from the sentiment of having once again failed, at protecting their community’s weakest, their women, children and elderly [27].

Despite its own set of limitations, this study is considered to be the first to provide such data, on the rates of trauma exposure, PTSD, and depression among individuals displaced to Iraqi Kurdistan’s refugee camps. Its main limitation pertains to sampling, which involved the recruitment of individuals consecutively visited by the social worker teams. Although the recruitment rate was high, not all the camp’s refugees had an equal probability of being interviewed by our teams. This bias could have influenced the various rates reported here. However, it is very important to note that, purposely, we did not include participants who were already followed for mental health problems, so as not to inflate such rates. Furthermore, the study did not sample refugees living outside the camps, as it is known, this specific population has had more socio-economic challenges than those residing inside the camps, due to a lack of systematized coverage from international and non-governmental organizations [28]. Therefore, we remain confident, that this study’s reported rates, do not overestimate the span of the problems observed.
